# Post-burn scars and scar contractures

**DOI:** 10.4103/0970-0358.70724

**Published:** 2010-09

**Authors:** Arun Goel, Prabhat Shrivastava

**Affiliations:** Departments of Burns, Plastic, Maxillofacial & Microvascular Surgery, Lok Nayak Hospital & Associated Maulana Azad Medical College, New Delhi – 110 002, India

**Keywords:** Burns, contractures, post-burn scars, release and cover

## Abstract

The mortality and morbidity from burns have diminished tremendously over the last six to seven decades. However, these do not truly reflect whether the victim could go back to society as a useful person or not and lead a normal life because of the inevitable post-burn scars, contractures and other deformities which collectively have aesthetic and functional considerations. This article gives an overview of the post-burn scars and scar contractures, especially their prevention, minimisation and principles of management.

## INTRODUCTION

Trauma is the leading cause of death and disability in the first four decades of life and the third most common cause of death overall. Burn trauma constitutes the second most common cause of trauma-related deaths after vehicular accidents, both in developing as well as the developed world. An extensive burn is the most devastating injury a person can sustain and yet hope to survive. Survival is no doubt the immediate concern, it is the restoration to pre-injury status, and return to society becomes important for the victim and the treating team. Burn survival statistics are definitely misleading in this. A healed burn patient may be left with scars have varying degrees of functional and aesthetic components.[1–3] Their actual incidence is not known. However, it is inversely proportional to the standards of initial treatment with patients receiving best of care having minimum number and severity of these problems.

### Post-burn scars

**Figure 1:**
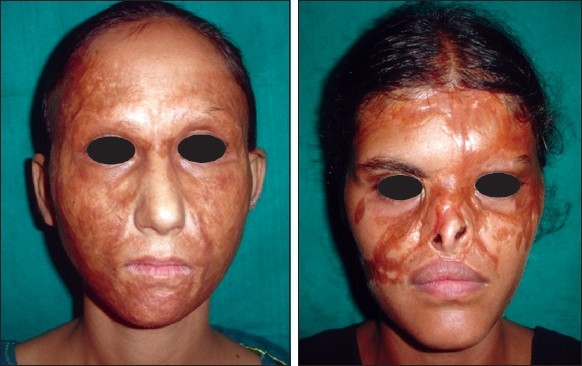
Post-burn scars involving whole of face (mature, soft and supple). Note hyperpigmentation and altered texture

**Figure 2:**
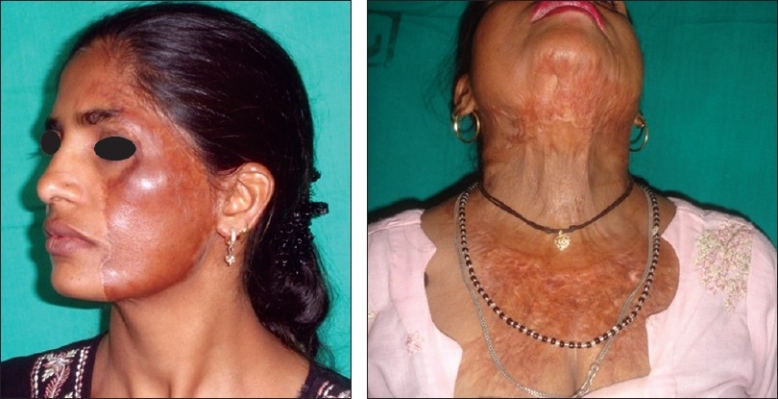
Excellent result after maturation of split-skin graft. From patient’s point of view, she is still “scarred”

Post-burn scars are inevitable even with the best of treatment because they depend upon the depth of burn injury. Except for the superficial dermal burns, all deeper burns (2nd degree deep dermal and full thickness) heal by scarring [Figure 1]. This scarring can only be minimised by various physical therapy measures and plastic surgical procedures but not eliminated completely. The appearance of even the best split-skin grafted areas and the donor sites of these grafts is also a “scar” by the patient’s definition of a scar [Figure 2].

### Post-burn scar contractures

**Figure 3:**
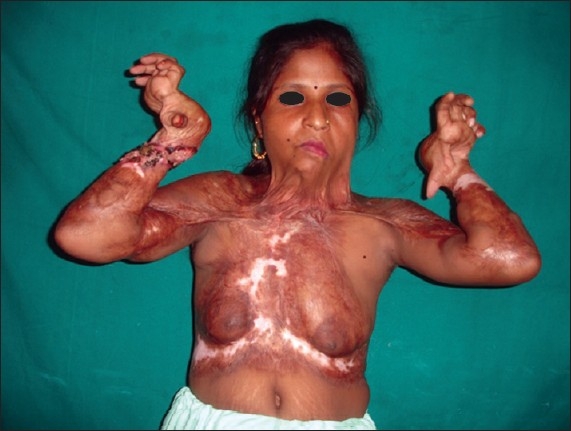
Multiple post-burn scar and scar contractures in an adult female

A burn patient who receives the best of treatment is expected to heal without any contractures.[4] The incidence of post-burn contractures is extremely high in our country. Quite often, they are not only multiple in a given patient but also very severe and diffuse. The number of trained burn and plastic surgeons is less than 1100 for more than 1100 million population in India. The patients are treated by a variety of service providers who aim at closing the raw wounds and this leads to invariable development wound contraction and scarring. Patients of post-burn contractures, defects and disfigurements constitute almost half of the workload of many general plastic surgeons, especially the ones in government institutions [Figure 3].

## BURN WOUND HEALING AND DEVELOPMENT OF SCAR AND SCAR CONTRACTURE

An understanding of the burn wound healing is fundamental not only to the management of the acute burn wound, but also for the prevention, minimisation and treatment of post-burn scars and scar contractures.[5,6] The healing of a burn wound is accomplished either by *restitution* (complete regeneration) or *substitution*. Restitution is possible only if the skin is burnt as deep as the stratum papillare and all the specialised cells of the organ are preserved. The epithelial cells, in these cases, are derived from the epithelial appendages such as pilosebaceous units and sweat glands in the central portion and wound edges at the periphery. These appendages extend into the deeper dermis and may even penetrate into the subcutaneous fat (as in beard area in males) and survive in partial thickness injuries. The sequence of cellular events that comprise epithelialisation include cellular detachment, migration, proliferation and differentiation. If the skin is affected deeper in the zone of stratum reticulare, the defect is covered by substitutive unspecialised connective tissue. The final result is demonstrated by a lesser or more extensive formation of the cicatrix. With full thickness loss of skin, wound contraction and epithelialisation from the margins occurs leading to contractures.

Contraction is an active biological process by which an area of skin loss in an open wound is decreased due to concentric reduction in the size of the wound. The reduction in size of wound causes lesser degree of connective tissue deposition and the amount of epithelialisation needed is decreased. Wound contraction involves an interaction of fibroblasts, myofibroblasts and collagen deposition and is a satisfactory mechanism when the tissue loss is small, in a non-critical area and surrounded by loose skin.

Scar contracture, on the other hand, is the end result of the process of contraction.

### Post-burn scars

**Figure 4:**
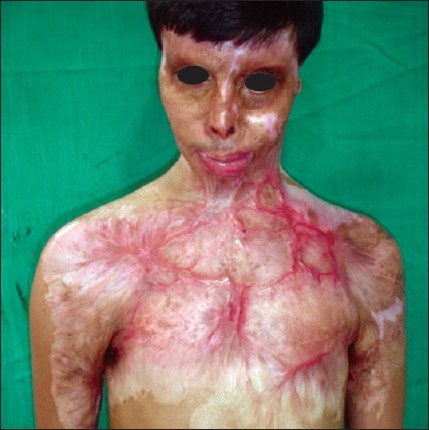
Post-burn contracture neck, bilateral axillae, ectropion left lower lid, lower lip and multiple vitiligo patches with loss of bilateral nipple areola complex (note the immature scars.

**Figure 5:**
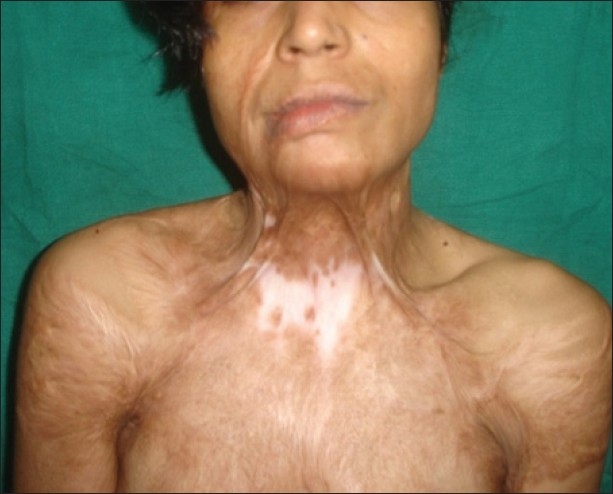
Post-burn contracture neck (note mature depigmented scars)

**Figure 6:**
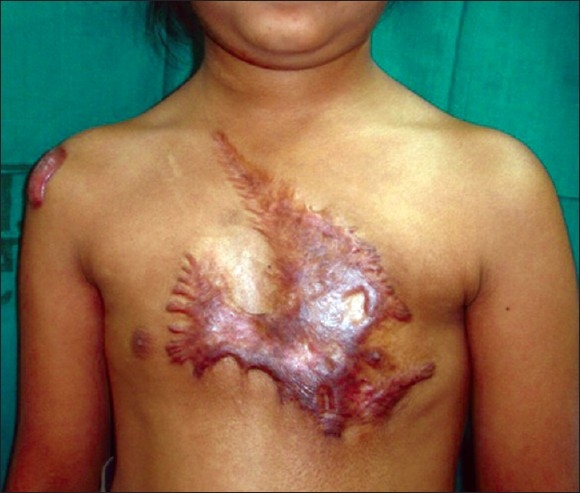
Post-burn hypertrophic scar on anterior chest wall.

**Figure 7:**
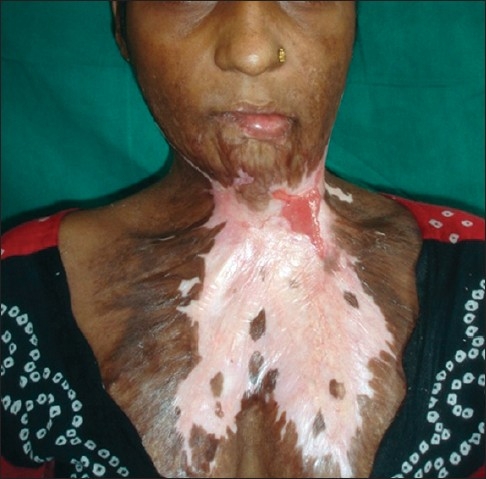
Post-burn contracture neck associated with raw areas and depigmented patches

**Figure 8:**
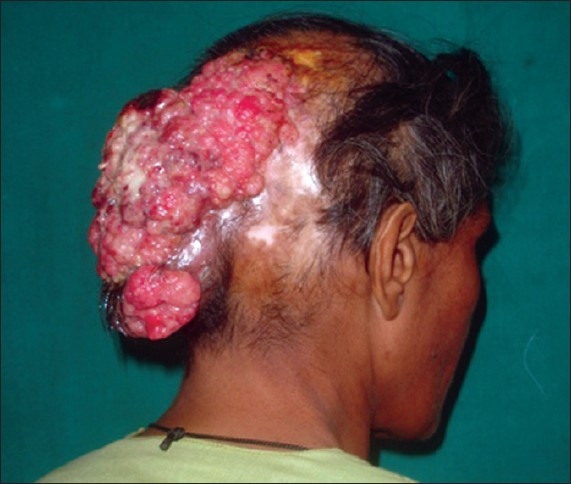
Post-burn scar of 18 years duration turning into malignancy (squamous cell carcinoma) – Marjolin’s ulcer

These may be immature/mature, atrophic/hypertrophic/ keloid, stable/unstable, depigmented (vitiligo)/hyperpigmented [Figures 4–7]. The scars may turn malignant as well [Figure 8]. As has been stated above, the post-burn scars are inevitable. Except for superficial burn injuries, all burn patients are bound to heal with scars, come what may. These scars on the skin are visible to the patient for the rest of his/her life and cause lifelong agony.

The extent and magnitude of scarring is directly related to the severity of burn injury. In a healed burn patient, the healing may have occurred either spontaneously with epithelialisation from remnants and contraction from wound margins or by split-skin grafting after early excision or over granulating raw areas after spontaneous eschar separation. In all these cases, the scar is immature and measures are taken to allow the scar to mature favourably.[7,8]

All the healed burnt areas as well as the donor sites tend to be dry and use of emollients is suggested and it helps in keeping the area supple and also reduces the burn itch. The overuse of lubricants is avoided as this may lead to furuncles.

Most of these patients complain of itching[9–10] on the healed burn and split-skin donor sites and need prescription of antihistaminic drugs (pheniramine maleate, promethazine, etc.) as well at least at bed time. Itching must be prevented not only to make the patient comfortable but also to break the cycle and ulceration. Gabapentin and its analogue, pregablin, have shown promising results in relieving this pruritis. Massage with steroid containing creams may also be advised for short periods on areas which can be protected from exposure to sunlight as otherwise hyperpigmentation can result.

The healed areas must also be prevented from exposure to sunlight till they are mature, to prevent hyperpigmention. This can be done by coverage with clothes, caps, sun screen lotions with SPF of at least 20–25, decreasing outdoor activity during daytime or even using an umbrella.

The favourable maturation of scars is also greatly helped by using custom-made pressure garments, especially for deep dermal burns and donor sites of intermediate and thick split-skin grafts which involve reticular dermis.[11–13] Very frequently, they develop hypertrophic scars. These unsightly scars are hyperaemic, raised above the surface of skin, firm to hard in consistency and itchy.

The compression garments prevent the development of hypertrophic scars. The effectiveness of these garments decreases after the development of hypertrophic scars. Before prescribing commercial pressure garments, the newly healed skin must be preconditioned to accept the stress and pressure exerted by the garments. For this, initially gentle pressure is applied with crepe bandages. As the skin toughens, the commercial pressure garments may be prescribed. These are to be worn 24 hours a day (except during bathing and massage) for a period of at least 9–12 months to be of benefit. In the hot and humid conditions of our country, it is difficult to wear them continuously and hence, compliance is a great problem, especially in children. Although this therapy has been in use for almost two centuries, the exact mechanism of action is not yet known. Pressure therapy diminishes the number of myofibroblasts, erythema, thickness and firmness of hypertrophic scar and accelerates its maturation. The various explanations put forward for these changes are hypoxia of the scar tissue by occlusion of the microvasculature, increased collagenase mediated collagen breakdown due to pressure induced decrease in capillary blood flow, reduction in tissue oedema, etc. The ideal pressure required is also not clear but it must be more than 24 mm Hg for capillary pressure to be countered.

**Figure 9:**
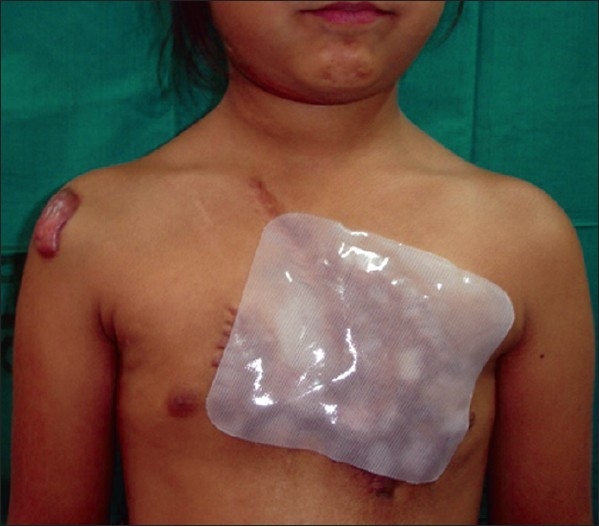
Post-burn hypertrophic scar being treated with Silicone Gel sheet

Silicone gel sheets have been shown to be useful for treatment of hypertrophic scars.[14] They have to be worn for 24 hours a day with particular care to be taken of local hygiene to avoid the development of contact dermatitis [Figure 9]. The exact mechanism of action of silicone gel is not known. It may exert its effects by increasing the temperature of the scar, thereby enhancing the activity of collagenase, which is known to increase several folds over 1–2°F rise of body temperature. Other effects of silicone gel, such as increased pressure, lowered oxygen tension and occlusion may be less important. Hydration of the stratum corneum and direct release of low molecular weight silicone fluid into the scar are other possible modes of action. However, silicone does not appear to enter scar tissues.

Intralesional injections of triamcinolone, every 4 weeks, have been found to be effective for control of hypertrophic scar. However, the dose and amount injected at any one time is limited and is useful for very small lesions only.

Hypopigmentation and hyperpigmentation following healing of burn injuries, whether spontaneously or with skin grafts, is very common although little is known about their causation. The depigmented or vitiligo patches are not only unsightly to the patient and the onlooker but also a taboo in our society, especially on areas like face, hands and feet, not covered by usual dress. The treatment involves excision/dermabrasion of vitiligo area and coverage with thin split thickness skin graft.[15] The colour may not match the surrounding area exactly but overall it blends with the surrounding scars and grafts leading to a satisfactory result. Very small areas may be tattooed with appropriate colours. Both hypopigmentation and hyperpigmentation patients are also advised use of cosmetics. A number of patients who do not get appropriate specialised treatment heal over prolonged periods of time by scar formation. This scar or neoepithelium is usually single-celled and highly fragile and bound to break due to minor trauma especially if present on extremities and other areas of stress (unstable scars). It needs excision/dermabrasion and coverage with intermediate thickness split-skin grafts. Any associated contracting element should also be released along with.

### Post-burn scar contractures

The scar may be supple or hypertrophic or keloidal. It may also show a tendency for repeated breakdowns. Any unstable area may also be associated with a Marjolin’s ulcer [Figure 8].

**Figure 10:**
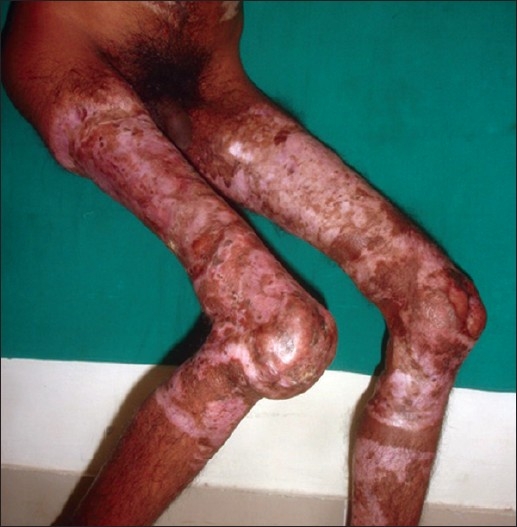
Bilateral post-burn contracture knee with subluxation of right knee joint

**Figure 11:**
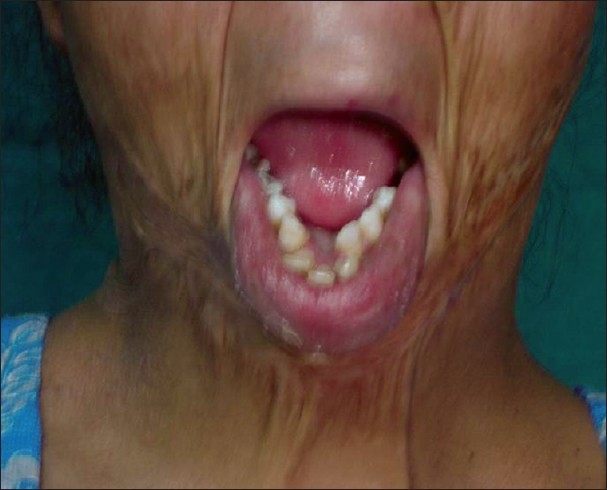
Post-burn contracture neck associated with deformed mandible

In addition, the deeper tissues may be affected either due to their involvement in the initial burn injury (e.g., electrical burns) or secondary to the presence of a skin contracture over a prolonged period of many years, which leads to shortening of musculotendinous units and neurovascular structures. The joints may be subluxated [Figure 10] or dislocated, with joint capsule and ligaments becoming tight in the direction of the contracture. The bones may be deformed, especially in growing children, e.g., mandibular deformity in cases of post-burn contractures of the neck [Figure 11]. Presence of one or more of above along with a contracture may alter the physical therapy and/or surgical treatment of a contracture. For example, an unstable scar or chronic non-healing ulcer(s) will not heal without surgical release of the contracture. Physical therapy prior to surgery may not be possible in these cases. Massive raw areas need wound closure with skin grafting before contracture can be subjected to physical therapy. Wide excision of Marjolin’s ulcer has to be combined with release of contracting bands. A post-burn contracture associated with a hypertrophic or an atrophic scar or a depigmented area may all need excision–release to achieve best results not only functionally but also aesthetically.

The shortening of deeper structures has great clinical significance. It may not be possible to release the contracture fully at the time of operation because the neurovascular structures and musculotendinous units may stand out as bowstrings, limiting any further release. Moreover, the vessels may also go into spasm with compromise of distal limb or digit circulation due to excessive stretch. The muscles/tendons may resist any lengthening by forceful pull even under general anaesthesia. Such contractures need gradual release by sustained traction using various methods for complete correction. This can generally be achieved within 2 weeks. Skin cover is provided only after complete release is achieved. In occasional circumstances, when the patient presents with severe post-burn contractures of elbow, wrist, knee, etc., decade(s) after sustaining the burn in childhood, it may not be possible to achieve complete release at all. When bones and joints are also affected, orthopaedic consultation may be needed. When mandible is deformed along with loss of normal dental occlusion in severe and untreated post-burn contractures of neck in children [Figure 11], orthodontic/ orthognathic measures are needed after the contracture is fully released.

The most important and effective method of controlling the wound contraction is to close the wound at the earliest using split-skin grafts in deep dermal and full thickness burns. Contraction can be inhibited by applying grafts to fresh wounds (as in early excision) or over healthy granulating areas (after eschar separation). Although full thickness skin grafts inhibit contraction almost completely, it is not possible in a clinical setting. The split-skin grafts may also need expansion with meshing in extensive burns. Although this leads to complete healing of the wound, the latter is largely covered with epithelium in the interstices of the meshed graft. It is widely believed that thicker the graft, greater will be the inhibition of contraction. This holds good only if the grafts are harvested from a given site. The fact is that when split-skin grafts are harvested from different sites, it is the total percentage of dermal thickness grafted which determines how much contraction will be inhibited. Delayed application of skin graft does not inhibit contraction as effectively as immediate grafting.

The scar collagen and elastin are relatively un-crosslinked and malleable during their initial deposition. Gentle, passive and sustained stretching exploits this malleability and is an effective technique for the lengthening of bands of scar tissue and increasing range of motion.

### Timing of surgery in post-burn contractures

As a general “rule”, surgical intervention for post-burn contractures should not be undertaken during the active phase of healing and scarring, i.e., as long as the scar is immature and highly vascular. This usually takes 1 year or so. One must allow the scar to become mature, soft and supple and “avascular” before undertaking surgery for contractures. This is because a highly vascular scar bleeds more during operation, with difficulties in achieving perfect haemostasis resulting in poor graft “take” leading to healing by further contraction. Secondly, operation on an active, highly vascular scar with wound bed still in active phase of contraction, adds insult to already traumatised tissues with vigorous local response in the form of further contraction. All these lead to a less than perfect result. Moreover, an immature scar is amenable to physical therapy measures resulting in significant improvement even non-surgically. With the passage of time, some mild contractures may improve with a better final result than if they had been surgically managed.

There are several exceptions to this general rule of scar maturation before doing surgical intervention.

- Ectropion of the eyelids, especially the upper eyelid with constant danger of keratoconjunctivitis, corneal ulceration, scarring or perforation with loss of vision
- Incapacitating contracture of the neck with inability to look forwards
- Severe microstomia causing interference with adequate nutrition and maintenance of orodental hygiene
- Crippling contractures of hand, especially dorsal contracture with metacarpophalangeal joints going in hyperextension leading to permanent damage to extensor mechanism with various deformities [Figure 3]
- Contractures of both the knees [Figure 10], which force the patient to be on “all the fours” and endangers the very dignity of being an upright human being
- Post-burn contractures with associated adjoining chronic raw areas needing skin cover
- Contractures with infected hypertrophic scars and abscesses, which need excision/drainage for their recovery
- Any severe, incapacitating contracture unlikely to improve at all with physical therapy measures

### Priority of release in cases of multiple contractures

A large percentage of patients coming to a burn surgeon have multiple contractures in different parts of the body. In addition, there may be other reconstructive needs, e.g., reconstruction of scalp, nose, pinna, face, etc.

Quite often, there may be limitations due to paucity of healthy donor sites for skin grafts and flaps. Therefore, a definitive strategy is essential in planning to reduce the number of anaesthetic and surgical procedures and yet provide the best functional and cosmetic result.

An *ectropion of the eyelids*, especially the upper eyelid, with its attendant risks needs to be dealt with first to prevent irreparable damage. One must remember that vision loss due to burns *per se* is rare expect for chemical burns. It may even be managed under local anaesthesia in the case of an adult. One eye should be operated at a time as operating on both the eyes together leads to temporary blindness due to dressings for a period of a week or so. This is highly distressing and frightening to any person with otherwise normal vision. However, if there is vision in only one eye, both eyes can be treated together.

Similarly, *a severe contracture of the neck or microstomia* leading to difficulties in intubation should be corrected before planning any other contracture release or reconstructive procedure requiring general anaesthesia. Additionally, the complete release of a neck contracture removes the extrinsic pull on facial structures (lips, chin and even lower eyelids) and contractures of the axilla and the breast.

*Dorsal contractures of the hand* need to be addressed after the neck is treated. They are not only crippling but may also cause permanent damage to the delicate balance of tendon movements (extensor apparatus) when metacarpophalangeal joints go into extension. Both hands should rarely be treated together (except in small children) to allow the patient his/her daily needs of feeding and toilet care. When axilla, elbow and hand are all affected on one side, it may not be possible to operate on the hand till the axilla and elbow are released.

*Contractures of the popliteal fossa* also need early intervention as they are also highly incapacitating.

## SURGICAL INTERVENTION

The surgical management of any post-burn contracture involves the following steps:

### Release of contracture

Complete release of contracture should be done, avoiding damage to any important underlying structure, e.g., arteries, nerves, tendons, etc. Although contraction occurs in all directions, the incision begins across the point of maximum tension, i.e., where the contracture is most tight. This point is usually opposite the joint line. The incision is deepened all the way to the unscarred tissues. Multiple darts should be made at appropriate points along the periphery of the defect created to take into account the contracture along other directions. Fishtailing of the incision line at either end is inadequate and inferior to the multiple darts. No attempt should ever be made at undermining the surrounding healthy/scarred skin and advancing it to decrease the defect. Incision line can be infiltrated with 1:200,000 adrenaline solution to have a relatively bloodless field. The limb contractures can be released under tourniquet which should be deflated after complete release and haemostasis achieved using bipolar cautery.

*Incision vs. excision:* In general, a contracture should be released by incision rather than by excision. This is especially true for patients who have received adequate pre-operative physical therapy and their scars have become soft and pliable. Incision alone decreases the requirement for skin cover. When the scars are extensive, it is futile to excise a small amount and all cannot be excised for fear of creating an extensive raw area. Excision may, however, be required in certain circumstances, e.g., (a) small adjoining depigmented or hypertrophic areas, excision of which will add to the final aesthetic result (b) atrophic/unstable scars/chronic non-healing ulcer(s)/ discharging sinuses should be excised along with release of contracture to obtain healthy bed for split-skin graft “take” (c) scars may also be excised so as to apply the graft/flap in accordance with principles of aesthetic units. Partial excision of hypertrophic scars may sometimes be done, e.g., in a case of post-burn contracture of neck, the scars may extend from chin, neck onto the chest and even abdomen. Here, only neck scars are excised.

**Figure 12:**
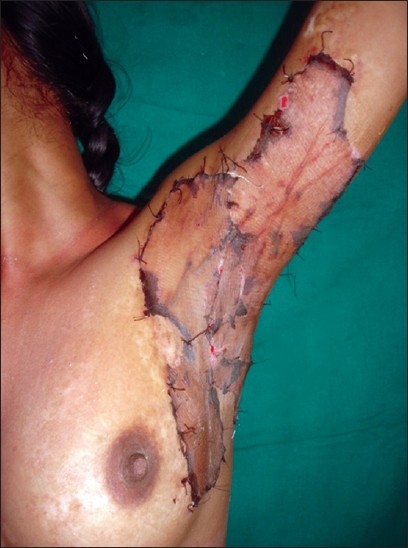
Post-burn axillary contracture being treated with sheet split-skin grafts

*Immediate vs. gradual release:* In general, the contracture should be released completely on the table in one go. However, in severe long-standing contractures, there is considerable shortening of musculotendinous units and neurovascular structures. Hence, it may not be possible to achieve complete release. Similarly, when the joints are subluxated or dislocated [Figure 12], immediate complete release may not be possible. In all these cases, as much release as possible is done and then, full correction is achieved gradually over a couple of weeks using serial splintage, skin/skeletal traction or the modern distractor systems (e.g., UMEX, JESS, etc). Once full correction is achieved, the skin cover is provided.

### Provision of skin cover

After the complete release of a post-burn contracture, the recreated defect has to be covered using skin grafts or a skin flap. Most commonly, the raw areas resulting after release of post-burn contractures are covered with skin grafts. Flap covers are used in special situations.

**Figure 13:**
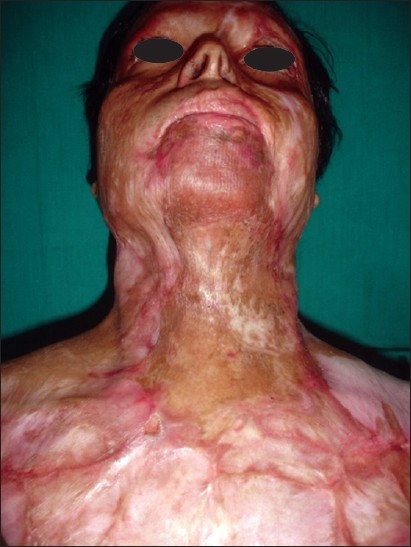
Post operative result after release and skin grafting of the patient in Figure 6

**Figure 14:**
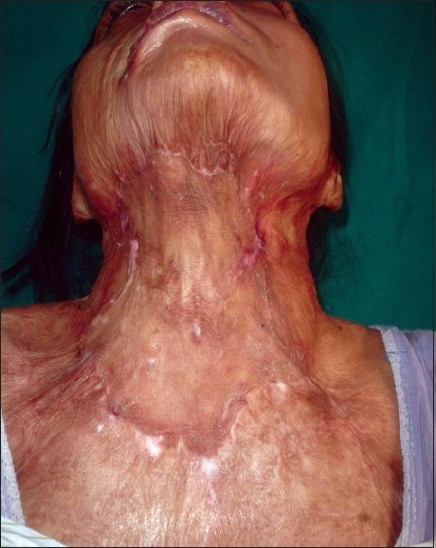
Post operative result after release and skin grafting of the patient in Figure 7

**Figure 15:**
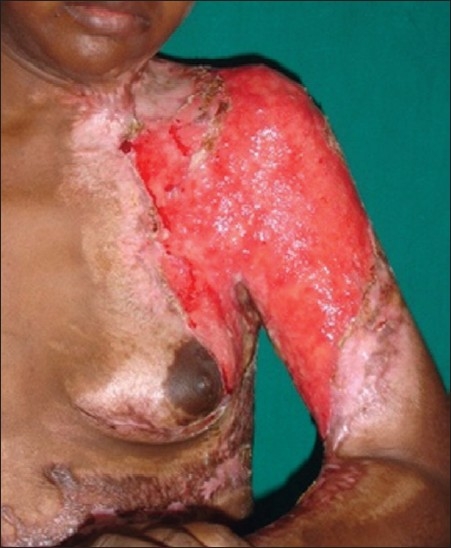
Multiple post-burn contractures with associated extensive raw area

**Figure 16:**
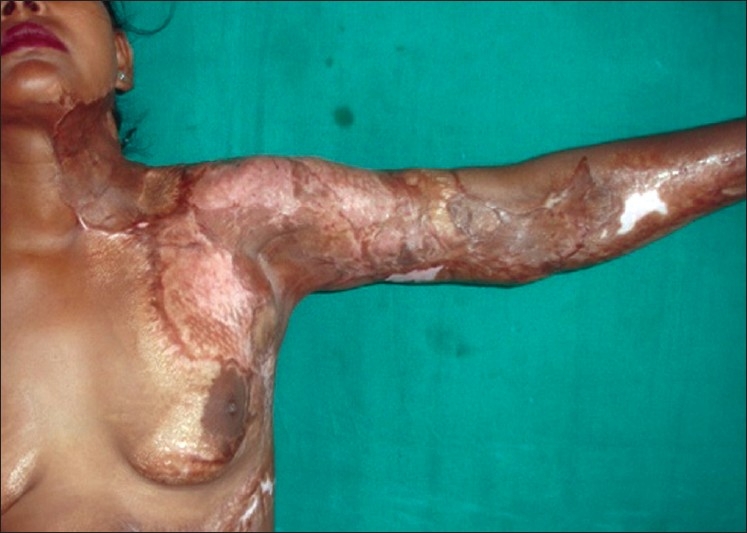
Post operative result of the patient in Figure 17

**Figure 17:**
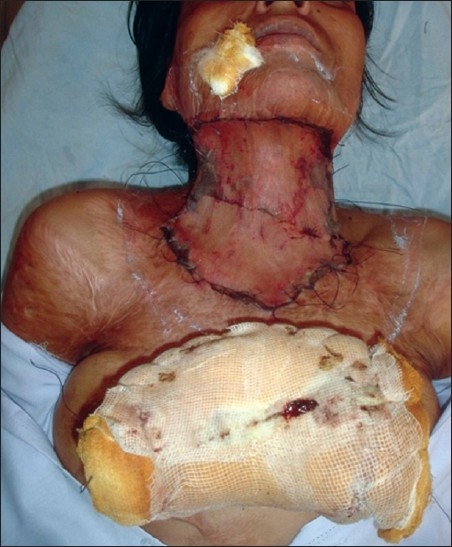
Sheet split-skin graft applied with tie over dressing with complete graft “take”

*Skin graft:*[16–18] When using grafts, sheet grafts are to be preferred and no attempts should be made to expand the graft by meshing [Figure 12]. As much as possible, try to feed in more graft than the size of the defect to take care of the postoperative, inevitable secondary contraction [Figures 4 and 13–16]. The junction line of the sheets of the grafts should be parallel to the axis of joint motion. The skin grafts are applied immediately after complete release. Sometimes, delayed application after 5 days, once the granulations form, is done. The delayed approach is required when release of contracture results in exposure of fat which is a poor bed for graft “take”. When the raw areas are oozing a lot, or when the contracture is associated with infected areas, delayed application can give a better result. When skin grafts have been used, they are immobilised by one or more of the various techniques, viz., “tie-over” dressings [Figure 17], plaster of Paris splints, crepe bandages, elastoplasts, etc., depending upon the site.

In general, all contractures should be treated with splitskin grafts of intermediate or thick variety [Figures 2b, 4b and 5c]. This allows the donor site to heal spontaneously. Moreover, extensively burnt patients with multiple contractures have a paucity of donor sites. Full thickness grafts, although better than the split-skin grafts in most of the properties, have poorer chances of “take” and their donor sites in turn need split-skin grafts. Their use is limited to very small defects resulting after release of contracture in aesthetically important areas, e.g., ectropion of the upper lip, lower eyelid, etc.

*Skin flaps:* There are a few situations where a skin flap is a must. If the contracture release is likely to open up the joint, especially of the hands and feet, or tendon/ nerve surgery is planned at a later date, e.g., old healed electrical burns, a flap cover must be provided after release of contracture. If release of contracture results in a moderate defect in a cosmetic area, which if covered with a flap will give a better aesthetic appearance, a flap cover may be provided. For example, an ectropion of the upper lip in an adult male can be released and covered with a flap from the scalp or upper neck to restore moustaches. In a female, the same ectropion requires a graft cover. A groin contracture may be treated using a tensor fascia lata flap with graft on the flap donor site. This decreases the need for postoperative splintage to prevent secondary graft contraction.

If a local flap in the form of Z plasty (for linear/webbed contractures), V-Y plasty, V-M plasty, etc., is available and will be sufficient in itself to treat the contracture, there is no reason why they should not be used. However, these flaps are always in danger of necrosis when raised in scar tissues. Moreover, they are possible only in very mild cases. They are also useful when massive diffuse contractures are treated with split skin graft (SSG) and later on, after graft maturation, contracting bands form at the graft-surrounding skin junction. The flaps used for provision of cover may be loco-regional or distant. They may be free or pedicled.

*Donor sites:* The split-skin grafts are usually harvested from thighs. However, in a severely burnt patient, with extensive scarring, the grafts may have to be harvested from legs, upper limbs and abdomen, scalp or back. For harvesting grafts from scalp, abdomen and back, electrical dermatome may be needed. It should be noted that in every patient who presents with a contracture, one must look for the donor sites available. In a case with multiple, massive contractures, the donor sites should be checked and a plan charted out for “which donor site for which contracture”. This is important as large sheets are usually required for neck, axilla and facial resurfacing, etc., while comparatively smaller pieces of grafts may be adequate for eyelid or finger contractures, etc.

### Postoperative care

*Maintenance of released/corrected position* is mandatory until the graft has become stable (usually 3 weeks) or till the flap margins have healed. Post-operative use of *static or dynamic splints*, interspersed with a routine of daily *physical therapeutic exercises* is required to keep the joints in full range of motion especially if static splintage is used. This therapy is continued till the grafts have matured and complete range of motion is achieved. Care of the grafted areas is done as detailed above till the graft loses its tendency to contract and can be pinched and moved over the recipient area (usually 1 year or so).

## References

[1] Hawkins HK, Pereira CT, Herndon DN (2007). Pathophysiology of the burn scar. Total Burn Care.

[2] Su CW, Alizadeh K, Boddie A, Lee RC (1998). The problem scar. Clin Plast Surg.

[3] Chavapil M, Koopmann C (1984). Scar formation: Physiology and pathological states. Otolaryngol Clin North Am.

[4] Schneider JC, Holavanahalli R, Helm P, Goldstein R, Kowalske K (2006). Contractures in burn injury: Defining the problem J. Burn Care Res.

[5] Cohen IK, Diegelmann RF, Lindblad WJ (1992). Wound Healing: Biochemical and clinical aspects.

[6] Fine NA, Mustoe TA, Greenfield LJ, Mulholland MW, Oldham KT, Zelenock GB, Lillemoe KD (2001). Wound Healing. Surgery: Scientific Principles and Practice.

[7] Poh-Fitzpatrick MB (1992). Skin care of the burned patient. Clin Plast Surg.

[8] Robson MC, Barnett RA, Leitch IO, Hayward PG (1992). Prevention and treatment of post burn scars and contracture. World J Surg.

[9] Casaer M, Kums V, Wouters PJ, Van den kerckhove E, Berghe GV (2008). Pruritis in patients with small burn injuries. Burns.

[10] Brooks JP, Malic CC, Jadkins KC (2008). Scratching the surface-Managing the itch associated with burns: A review of current knowledge. Burns.

[11] Cara Collins JA (1992). Pressure techniques for the prevention of the hypertrophic scar. Clin Plast Surg.

[12] Sawada Y (1993). Pressure developed under pressure garments. Br J Plast Surg.

[13] Macintyre L, Baird M (2006). Pressure garments for use in the treatment of hypertrophic scars: A review of the problems associated with their use. Burns.

[14] Kerckhove EV, Stappaerts K, Boeckx W, Hof BV, Monstrey S, Kelen AV (2001). Silicones in the rehabilitation of burns: A review and overview. Burns.

[15] Grover R, Morgan BD (1996). Management of hypopigmentation following burn injury. Burns.

[16] Harrison CA, MacNeil S (2008). The mechanism of skin graft contraction: An update on current research and potential future therapies. Burns.

[17] Iwuagwa FC, Wilson D, Bailie F (1999). The use of skin grafts in burn contracture release: A 10-year review. Plast Reconstr Surg.

[18] Rudulph R, Ballantyne DL, McCarthy JG (1990). Skin Grafts. Plastic Surgery.

